# Drug Utilization, Anticholinergic Burden and Predictors of Length of Stay in a Psychiatric Hospital: A Retrospective Observational Study

**DOI:** 10.3390/medicina62061063

**Published:** 2026-05-31

**Authors:** Zeliha Arzu Özdemir Sincar, Elif Ertuna, Öznur Altıparmak, Ayşegül Koç, Mehmet Zuhuri Arun

**Affiliations:** 1 İzzet Baysal Mental Health and Diseases Hospital, Bolu 14300, Turkey; 2 Department of Clinical Pharmacy, Faculty of Pharmacy, Ege University, İzmir 35040, Turkey

**Keywords:** antipsychotic polypharmacy, drug utilization, drug interactions, clinical pharmacy, length of stay

## Abstract

*Background and Objectives*: Mental illnesses place a substantial burden on healthcare systems and often require complex pharmacological management. However, it remains unclear whether early antipsychotic polypharmacy independently predicts length of stay after adjusting for important confounders. This study aimed to investigate drug utilization patterns, including polypharmacy and drug–drug interactions (DDIs), assess anticholinergic burden, and identify predictors of the length of hospital stay in a psychiatric inpatient setting. *Materials and Methods*: This retrospective, observational study was conducted at Bolu İzzet Baysal Mental Health and Diseases Hospital with 280 adult patients admitted during 2022. Medication data were extracted from electronic medical records based on medication orders within the first 72 h of admission. Potential DDIs were assessed using Lexi-Interact, and anticholinergic burden was calculated using the ACB, ADS, and ARS scales. Predictors of the length of stay (LOS) were modelled using negative binomial regression. *Results*: The mean age of the population was 38.65 ± 13.86 years, and 62.1% were male. Polypharmacy was present in 37.9% of patients, while antipsychotic polypharmacy was observed in 63.9%. Potential DDIs were identified in 88.9% of patients, with a significantly higher prevalence in those with polypharmacy. Mean ACB and ADS scores were high at 5.56 ± 3.32 and 4.15 ± 2.79, respectively. Multivariable regression analysis revealed that antipsychotic polypharmacy was the primary independent predictor of prolonged hospitalization, associated with a 20.9% increase in LOS (IRR = 1.209, 95% CI: 1.044–1.400, *p* = 0.011). While age was also statistically significant (IRR = 1.006, 95% CI: 1.001–1.012, *p* = 0.019), its clinical impact was minimal, representing only a 0.6% increase in LOS per year. *Conclusions*: Antipsychotic polypharmacy within the first 72 h of admission is a significant independent predictor of prolonged hospitalization. The high prevalence of drug interactions and substantial anticholinergic burden highlight the need for systematic early medication review. Instead of general monitoring, targeted medication therapy review focusing on antipsychotic polypharmacy in the early period of admission may be essential to identify and mitigate modifiable risk factors for prolonged hospitalization, thereby optimizing pharmacotherapy in psychiatric inpatient settings.

## 1. Introduction

Mental disorders represent a significant global public health challenge, substantially reducing patient quality of life, imposing a heavy burden on healthcare systems [1,2]. Pharmacological treatment, along with psychotherapy, is considered a fundamental approach to the management of these illnesses [3]. Therefore, optimizing drug utilization is essential for successful clinical outcomes in psychiatry.

In psychiatric hospitals, management of severe and complex cases often necessitates intensive pharmacological treatment, which inevitably leads to polypharmacy. Generally defined as the concurrent use of 5 or more medications, polypharmacy prevalence in psychiatric settings ranges from 13% to 90% [4,5,6]. Specifically, antipsychotic polypharmacy is the use of 2 or more antipsychotic drugs at the same time [7]. It has been estimated that approximately 15–40% of patients have antipsychotic polypharmacy [8]. Recent literature indicates that psychiatric polypharmacy most commonly emerges in patients with treatment-resistant and severe clinical presentations. In particular, within schizophrenia spectrum disorders, insufficient response to monotherapy often leads clinicians to adopt antipsychotic polypharmacy, with studies reporting that a substantial proportion of these patients receive multiple psychotropic medications concurrently [9]. A major clinical consequence of such complex regimens is the heightened risk of drug–drug interactions (DDIs), which can increase up to 3.7-fold in the presence of polypharmacy [10].

Beyond the risk of DDIs, an important pharmacodynamic concern in psychiatry is the anticholinergic burden. This arises from the cumulative use of multiple medications with anticholinergic activity, including antipsychotics, tricyclic antidepressants, and antiparkinsonian agents. To quantify the anticholinergic risk, several scales have been developed. One of the most widely used tools is the Anticholinergic Cognitive Burden (ACB) Scale, introduced by Boustani et al. in 2008 [11,12], which focuses on the relationship between anticholinergic use and cognitive decline. The other two prominent scales are the Anticholinergic Risk Scale (ARS) and Anticholinergic Drug Scale (ADS), developed by Rudolph et al. and Carnahan et al., respectively [13,14]. Given that an elevated anticholinergic burden is known to have a negative effect on the neurocognitive performance of patients, its assessment is particularly important in psychiatry [12]. In addition to these cognitive impairments, high cumulative anticholinergic exposure often results in severe peripheral and multi-systemic complications. These complications include acute autonomic dysregulation, such as tachycardia, urinary retention, blurred vision, and significant gastrointestinal hypomotility, which may progress to severe constipation or paralytic ileus. Systematic evidence indicates that clinicians should exercise caution to avoid inappropriate prescribing of these medications, given their broad range of central adverse effects, including dizziness, sedation, confusion, and potential delirium. These effects collectively contribute to declines in both cognitive and physical functioning [15].

Ultimately, these complex pharmacological factors may influence broader clinical outcomes, such as the length of hospital stay (LOS). While non-pharmacological factors like age, previous hospitalizations, and having received electroconvulsive therapy are known predictors of LOS [16], drug-related factors, particularly antipsychotic polypharmacy, also play a significant role [7,17].

While polypharmacy and drug–drug interactions (DDIs) are frequently reported in psychiatric settings, it remains unclear whether early medication patterns specifically antipsychotic polypharmacy established within the initial 72 h of admission can independently predict the length of hospital stay (LOS). Although previous studies have evaluated general prescription patterns and factors associated with DDIs in psychiatric patients, there is still a lack of consensus on how these early clinical decisions influence hospitalization duration [18,19]. Furthermore, while the cumulative anticholinergic burden is a significant concern in psychiatric pharmacotherapy, it is not documented in acute inpatient settings.

Therefore, this study aimed to evaluate early drug utilization patterns in a psychiatric hospital in Türkiye, focusing on prescribing characteristics, polypharmacy, potential DDIs, and anticholinergic burden, while identifying key predictors of length of hospital stay.

## 2. Materials and Methods

### 2.1. Study Design

This single-centre, retrospective, observational study was conducted at İzzet Baysal Mental Health and Diseases (IBMHD) Hospital, Türkiye. IBMHD is a 105-bed specialized psychiatric facility serving seven provinces in the Western Black Sea region. The hospital operates six inpatient wards, five of which are closed (locked) units designed for intensive clinical monitoring and crisis stabilization. A significant portion of the patient population in these units (approximately 40–45%) consists of forensic cases. The study protocol was first approved by the İzmir Tınaztepe University Ethics Committee (Date: 13 December 2023; No: 2023/38), followed by official permission from the Chief Physician of the psychiatric hospital. The authors complied with Good Clinical Practice standards throughout the study. All procedures were performed in accordance with the ethical principles of the Declaration of Helsinki.

### 2.2. Patient Selection

Hospital records were screened to identify adult patients admitted to the inpatient wards between 1 January 2022, and 31 December 2022. From the initial pool of eligible patients, 280 individuals were selected using a simple random sampling method via SPSS software (IBM SPSS Statistics for Windows, Version 25.0; IBM Corp., Armonk, NY, USA), where each patient was assigned a unique sequential number based on their admission order. In cases where a selected patient did not meet the inclusion criteria, a new patient was randomly selected to maintain the target sample size. The inclusion criteria for the study were being 18 years of age or older, being admitted to inpatient ward for more than 72 h, and using at least one medication during the hospital stay. Patients were excluded from the study if their medical records were incomplete, contained inconsistent data, lacked a definitive diagnosis, or if official authorization to access their clinical records was unavailable.

### 2.3. Data Collection

Clinical data were extracted from electronic medical records, encompassing demographics (age and sex), comorbidities. Medication data were collected within the first 72 h of each patient’s admission. This assessment window was selected based on the early in-hospital medication review framework established by Hohl et al., which defined early medication review as an intervention initiated within 24 h of emergency department presentation or within 72 h of hospital admission [20]. For patients with multiple admissions during the retrospective screening period, only the index hospitalization was included in the study. Standardized case report forms were used for data collection. The study database was constructed in SQLite, with a specific drug table built using the registered drug list from the Turkish Medicines and Medical Devices Agency. All data were recorded through custom-developed software (developed by MZA) and subsequently retrieved for analysis using structured SQL queries.

The reasons for hospitalization were grouped into seven main categories based on ICD-10 main groups: alcohol/substance use disorders, anxiety disorders, mood disorders, personality disorders, psychotic disorders, intellectual disabilities, and others. This clinical grouping was performed by an experienced psychiatrist.

Potential DDIs were assessed for each patient using the Lexi-Interact module of the UpToDate drug database (www.uptodate.com). Only clinically significant DDIs (Lexi risk ratings C, D, and X) were recorded. For each identified DDI, the risk rating, severity, and reliability ratings were also documented.

The anticholinergic burden of each patient’s medication regimen was assessed using three validated scales: the Anticholinergic Cognitive Burden (ACB) scale [11], the Anticholinergic Drug Scale (ADS) [13], and the Anticholinergic Risk Scale (ARS) [14].

### 2.4. Statistical Analysis

An a priori sample size calculation was performed to determine the minimum required number of participants for the study. Given a total population of 781 unique patients admitted during the study period, the minimum sample size was calculated as 258, assuming a 95% confidence level and a 5% margin of error. To account for potential data loss or exclusions, a 10% reserve was added, and 290 patients were initially selected. Following a rigorous review of clinical records, 10 patients were excluded due to incomplete or inconsistent electronic medical records. Consequently, the final statistical analyses were conducted with 280 patients.

The normality of continuous variables was assessed using the Shapiro–Wilk test alongside skewness and kurtosis coefficients, with values within ±2 considered acceptable, and further confirmed by visual inspection of histograms and Q-Q plots. Homogeneity of variances was assessed using Levene’s test.

Parametric and non-parametric continuous variables were expressed as means ± standard deviation (SD) or median (interquartile range–IQR), where appropriate. Categorical data were presented in terms of frequencies. The independent-samples *t*-test or Mann–Whitney U-test was used for comparisons between the subgroups of continuous variables. Categorical data were evaluated using Chi-Square test. Correlations were assessed using Pearson’s correlation coefficient for normally distributed variables and Spearman’s rank correlation coefficient for non-normally distributed variables.

Given the count nature and over dispersed distribution of the length of hospital stay (variance/mean ratio = 6.81), negative binomial regression was used to model predictors of length of stay. Univariate negative binomial regression was first performed for each candidate predictor (Table S1). Collinearity was assessed using variance inflation factors (VIF), and collinear variables were not included simultaneously. Results were reported as incidence rate ratios (IRR) with 95% confidence intervals (CI). Model fit was assessed using the Akaike Information Criterion (AIC), Bayesian Information Criterion (BIC), and likelihood ratio tests. Robust standard errors were used in negative binomial regression model.

Biperiden was not included as an independent predictor in the regression model because it is prescribed to manage antipsychotic-induced extrapyramidal side effects and therefore represents a pharmacological consequence of antipsychotic use rather than an independent prescribing decision. Its contribution was instead assessed through the anticholinergic burden analysis.

Statistical analyses were performed using SPSS 25. Regression analyses were conducted using Python 3.11 with the pandas, scipy, and statsmodels libraries in a Jupyter Notebook version 5.9.1. A two-tailed *p*-value < 0.05 was considered statistically significant.

## 3. Results

### 3.1. Demographic and Clinical Characteristics of Patients

A total of 280 patients were included in the study. Male patients accounted for 62.1% of the study population. The mean age of the study population was 38.65 ± 13.86 years, with no statistically significant difference between males and females. Only 13 patients were classified as geriatric (≥65 years) (Table 1).

The most frequent reasons for hospitalization were psychotic disorders and mood disorders. Examination of clinical records indicated that, 21.4% (*n* = 60) of the patients had at least one comorbidity; of these, hypertension (8.6%) and diabetes mellitus (7.9%) were the most prevalent (Table 1).

### 3.2. Drug Utilization

Drug utilization characteristics of the patients are summarized in Table 2. The median number of drugs per patient was 4 (IQR: 3–5, range: 1–17). Polypharmacy (≥5 drugs) was present in 106 patients (37.9%). Patients with comorbidities had a significantly higher number of drugs compared to those without (median: 6 vs. 3; Mann–Whitney U, *p* < 0.001). A clinically very weak but statistically significant positive correlation was observed between age and the total number of drugs (Spearman’s *rs* = 0.149, *p* = 0.012), indicating that the statistical significance is likely driven by the sample size rather than a robust clinical effect.

**Figure 1:**
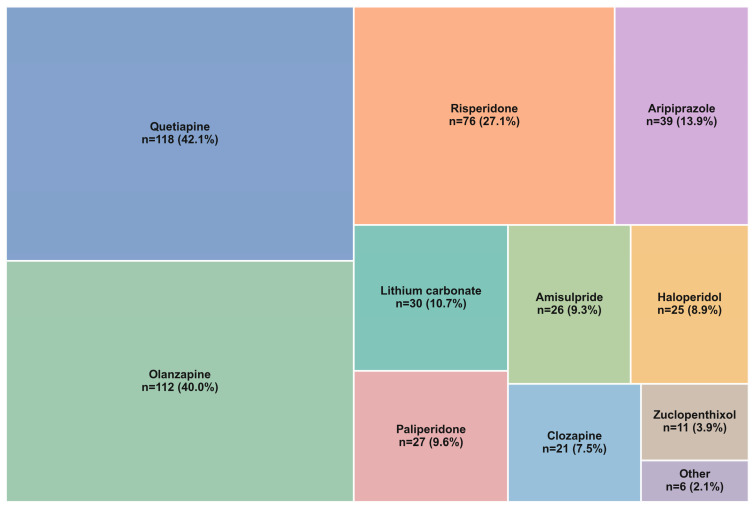
Distribution of antipsychotic drugs. Other drugs were chlorpromazine (*n* = 3), sulpiride (*n* = 2) and trifluoperazine (*n* = 1).

At least one antipsychotic drug was prescribed to 263 patients. Antipsychotic polypharmacy, defined as the use of multiple antipsychotics, was prevalent in 179 (63.9%) patients. Atypical antipsychotics were markedly more commonly prescribed than typical antipsychotics. The most frequently prescribed antipsychotics were quetiapine, olanzapine and risperidone. The rate of antipsychotic polypharmacy was significantly lower in geriatric patients compared to younger adults (30.8% vs. 68.5%; χ^2^ = 5.080, *p* = 0.024) (Figure 1; Table 2).

A total of 31.4% of patients were prescribed at least one antidepressant. The most frequently prescribed pharmacological group was SSRIs (*n* = 61 prescriptions), followed by SNRIs (*n* = 28). Sertraline (*n* = 36 patients) and venlafaxine (*n* = 18 patients) were the most commonly prescribed individual antidepressants. Biperiden, an anticholinergic agent used for the management of extrapyramidal symptoms, was prescribed to 63 patients (22.5%).

Most frequently prescribed drug classes other than antipsychotics and antidepressants are summarized in Table 3. Mood-stabilizing antiepileptics, primarily valproic acid (23.6%) and carbamazepine (5.7%) were also commonly used. Biperiden was prescribed to 22.5% of patients. Concomitant medications, including thyroid hormones (6.1%), beta-blockers (6.8%), and antihypertensives (4.6%), were consistent with the comorbidity profile of the study population.

### 3.3. Drug–Drug Interactions

A total of 1305 potential drug–drug interactions (DDIs) were identified, with at least one interaction detected in 88.9% (*n* = 249) of the patients. The median number of DDIs per patient was 4 (IQR: 1–7, range: 0–34). According to risk category, the majority of DDIs were categorized as risk rating C (*n* = 1207, 92.5%), followed by category D (*n* = 84, 6.4%) and category X (*n* = 14, 1.1%). The most frequently identified DDIs for each risk category are presented in Table 4.

The number of potential DDIs was significantly higher in patients with polypharmacy, antipsychotic polypharmacy, and biperiden use (Table 5). Based on the median DDI count, the high DDI group (>4), included 78.2% of patients with polypharmacy (χ^2^ = 113.908, *p* < 0.001), and 61.0% of those with antipsychotic polypharmacy (χ^2^ = 36.956, *p* < 0.001).

### 3.4. Anticholinergic Burden

The mean ACB and ADS scores were 5.56 ± 3.32 and 4.15 ± 2.79, respectively; the median ARS score was 2 (IQR: 1–3). All three scores were significantly higher in patients with antipsychotic polypharmacy (Table 6). Strong positive correlations were observed between anticholinergic burden scores and the number of DDIs (ACB: Spearman’s *rs* = 0.625, *p* < 0.001; ADS: *rs* = 0.500, *p* < 0.001; ARS: *rs* = 0.442, *p* < 0.001). However, no significant correlation was found between anticholinergic burden scores and the length of hospital stay (ACB: Pearson *r* = 0.065, *p* = 0.275; ADS: Pearson *r* = 0.072, *p* = 0.228; ARS: Spearman *rs* = 0.092, *p* = 0.124).

### 3.5. Factors Associated with Length of Hospital Stay

Bivariate comparisons of length of hospital stay are presented in Table 7. Neither the presence of medical comorbidities nor polypharmacy effected length of stay (*p* = 0.515 and *p* = 0.063, respectively). In contrast, patients with antipsychotic polypharmacy had a significantly longer hospital stay (23.03 ± 12.24 vs. 19.23 ± 11.64 days; *p* = 0.012). Presence of psychotic disorder was associated with longer stay (24.50 ± 12.80 vs. 19.05 ± 11.22 days; *p* < 0.001), whereas mood disorder was associated with shorter stay (19.50 ± 10.80 vs. 23.22 ± 12.70 days; *p* = 0.009).

**Figure 2:**
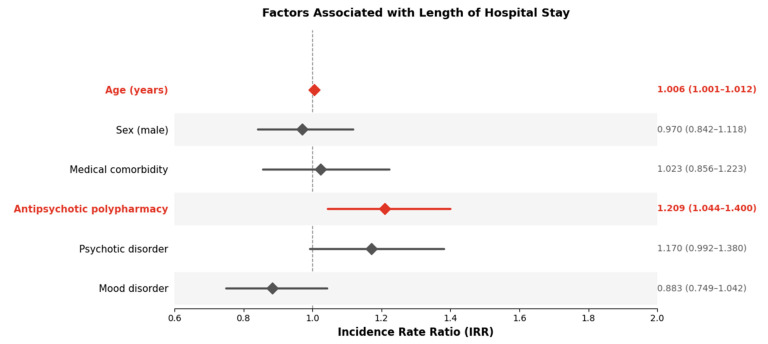
**Forest plot of negative binomial regression model for length of hospital stay.** Diamond markers represent incidence rate ratios (IRR) and horizontal lines indicate 95% confidence intervals. The dashed vertical line represents the null value (IRR = 1.0). Red indicates statistically significant predictors (*p* < 0.05).

Given the over-dispersed distribution of the length of hospital stay, a negative binomial regression model was constructed (Table 8, Figure 2). Antipsychotic polypharmacy was independently associated with a 20.9% increase in the length of hospital stay (IRR = 1.209, 95% CI: 1.044–1.400, *p* = 0.011). Age was also a significant predictor (IRR = 1.006, 95% CI: 1.001–1.012, *p* = 0.019).

## 4. Discussion

This study investigated drug utilization patterns and related parameters, specifically polypharmacy, anticholinergic burden, and drug–drug interactions and analysed their association with the length of hospital stay among 280 psychiatric inpatients in Türkiye. Our key findings summarized in Table 9.

The patient population in our study was relatively young, predominantly male, with a low proportion of geriatric patients. This demographic profile is consistent with large-scale studies of psychiatric inpatients. Toto et al. [21] reported a similar profile in a multicentre study of approximately 31,000 psychiatric inpatients across five European countries. Due to the relatively young age of the majority of patients, the number of medical comorbidities and, consequently, the use of non-psychiatric medications, such as antihypertensives, were low. This may indicate that medication experience in these wards is focused on the treatment of mental disorders and the management of adverse effects associated with psychotropic medications. Clinical pharmacy services provided by pharmacists in these wards may also support the healthcare team’s drug knowledge when medications from different therapeutic classes are required.

During hospitalization, the presence of prominent positive symptoms in schizophrenia patients and the presence of hostile/aggressive behaviour have been identified as factors for the application of antipsychotic polypharmacy [22]. In a multicentre study conducted on hospitalized psychiatric patients, the rate of using three or more antipsychotic drugs was reported as 44.7% [21]. The benefit rate of antipsychotic polypharmacy may be especially higher in patients who require intensive treatment. A study conducted on inpatients in Finland showed that antipsychotic polypharmacy did not increase the risk of mortality [23]. However, a more recent large-scale population-based study indicated that antipsychotic polytherapy was associated with a 17% increase in the risk of all-cause mortality, suggesting that the synergistic effects of multiple medications may create a clinical vulnerability that transcends simple dosage considerations [24]. On the other hand, it may be a significant risk factor for the occurrence of adverse effects. In a retrospective cohort study conducted in Japan, the frequency of antipsychotic polypharmacy among inpatients was reported as 24%, and it was found that the risk of adverse events increased in these patients [25].

It is known that the frequency of atypical antipsychotic polypharmacy may vary from country to country. In North America, it has decreased over the years from around 55% to 19%. In Europe, the median is around 24%. However, since most of these studies cover outpatients, the prevalence rate obtained in our study may not be consistent with these findings [26]. In our study, antipsychotic polypharmacy was present in 63.9% of patients using antipsychotic drugs. One reason for this rate being higher than those reported in the literature may be that the secondary-level regional hospital where the study was conducted serves a wider area, particularly handling psychiatric emergency cases. It is accepted that, in psychiatric clinics, patients who require hospitalization generally have a more severe course of illness [27]. Although current clinical guidelines continue to recommend monotherapy as the first-line approach, recent systematic reviews and meta-analyses suggest that selected combinations may provide clinical benefits in resistant cases, albeit with an increased risk of adverse effects and drug interactions [28]. In this study conducted on inpatients, the frequent occurrence of antipsychotic polypharmacy may be due to the patients’ need for more intensive medication treatment.

Among patients who received antipsychotic polypharmacy during inpatient treatment, a high dose requirement for antipsychotics and/or continued antipsychotic polypharmacy is more frequently observed at discharge [22]. Additionally, patients discharged with antipsychotic polypharmacy have higher rates of rehospitalization within six months [29]. Therefore, during the discharge process for these patients, the treatment applied during hospitalization should also be taken into account when providing medication education to patients and caregivers and when organizing clinical pharmacy services for treatment management.

A prolonged hospital stay is a determining factor for antipsychotic polypharmacy at the time of discharge [30]. In another study, a regression model demonstrated that each additional antipsychotic drug added to the treatment increased the length of hospital stay by approximately 6.56 days [4]. The key finding of our study is that antipsychotic polypharmacy was independently associated with a 20.9% increase in the length of hospital stay in the negative binomial regression model (IRR = 1.209, *p* = 0.011), which is consistent with these findings. To the best of our knowledge, this is the first study from Türkiye to demonstrate that antipsychotic polypharmacy independently predicts prolonged hospitalization using a count regression model. Furthermore, a novel methodological aspect of our study is that the assessment of antipsychotic polypharmacy was based on medication orders placed within the first 72 h of admission, thereby capturing the acute treatment decisions that may influence the subsequent clinical course. Identifying antipsychotic polypharmacy within the first 72 h of admission may provide a strategic advantage for clinicians to recognize patients at risk for prolonged hospitalization and those requiring intensified medication therapy management. A dedicated sex-stratified analysis or inclusion of an interaction term was not incorporated into the final regression model. Future research may yield more definitive findings by utilizing larger datasets that include additional parametric variables, such as gender-specific modulators.

Medical comorbidity was not a significant predictor of length of stay in our regression model. Consistent with our findings, Douzenis et al. reported that medical conditions typically prolong psychiatric hospitalization only when they are active or severe enough to require specialist referral [31]. Although medical referrals or the acute severity of medical comorbidities was not assessed in our study, given the relatively young age of our patients most comorbidities were likely stable chronic conditions. In such cases, these comorbidities might not have necessitated acute medical intervention, allowing the discharge process to be governed primarily by psychiatric stabilization rather than medical complexity. Consequently, in this specialized inpatient setting, length of stay appears to be driven more by psychiatric symptom severity and pharmacotherapy factors—such as antipsychotic polypharmacy—rather than stable physical comorbidities.

Psychotic disorder was a highly significant predictor of longer length of stay in bivariate analysis. However, this association was reduced to borderline significance in the multivariable model (IRR = 1.170, *p* = 0.062). This reduction is likely due to the shared variance between psychotic disorder and antipsychotic polypharmacy. Patients with psychotic presentations exhibited the highest rates of antipsychotic polypharmacy and biperiden use. Inclusion of antipsychotic polypharmacy in the model accounted for a substantial portion of the variance previously attributed to psychotic diagnosis alone. These findings indicate that the extended hospitalization observed in patients with psychotic disorders is partially mediated by more intensive antipsychotic pharmacotherapy, rather than being solely attributable to the underlying diagnosis.

Our study found no association between polypharmacy and length of stay, indicating that the conventional numerical threshold for polypharmacy may not be the most relevant measure for early psychiatric inpatient care. In this clinical context, the specific characteristics of medication regimens such as antipsychotic polypharmacy seem to influence clinical outcomes and resource use more than the total number of drugs. Mohamed et al. similarly reported that certain antipsychotic combinations and high-dose patterns were more closely linked to adverse effects than simply the number of medications taken [18]. Further research is needed to develop psychiatric-specific definitions of polypharmacy.

Safe medication use is the most important concern in the treatment of mental disorders [32]. Patients often require complex treatments, and medication-related problems are common. Among hospitalized psychiatric patients, the most frequently encountered medication-related problem is drug interactions, and pharmacists play a significant role in identifying and managing these interactions [33,34]. In our study, at least one drug interaction was observed in a significant portion of patients.

It has been reported that the risk of drug–drug interactions and the incidence of adverse events related to increased drug–drug interactions are higher in patients with antipsychotic polypharmacy and/or general polypharmacy [10]. Pharmacists can play a significant role in the identification and management of drug–drug interactions among patients in psychiatric hospitals [35]. The high number of detected drug interactions in patients with antipsychotic polypharmacy can be considered an important issue that pharmacists should pay close attention to in clinical pharmacy services for the management of these patients’ treatment.

A high prevalence of vitamin B combination use was observed in our study. This can be partly explained by the role of B vitamins as essential coenzymes in the synthesis of monoamine neurotransmitters, providing adjunctive support to optimize psychotropic responses [36]. Moreover, acute psychiatric inpatients often present with severely unhealthy dietary habits and poor self-care prior to admission; consequently, prompt vitamin restoration becomes a common clinical practice [37]. In addition, psychiatric patients who use certain medications such as PPIs and lithium, which can induce iatrogenic malabsorption, carry a significantly higher risk for developing vitamin B deficiencies [38]. Taken together, these clinical rationales may explain the widespread use of vitamin B combinations in acute psychiatric inpatient settings.

Biperiden is a potent anticholinergic drug commonly utilized to manage extrapyramidal symptoms (EPS) associated with antipsychotic medication use [39]. A total of 63 patients (22.5%) were prescribed biperiden for the management of antipsychotic-induced extrapyramidal symptoms. In our study, patients prescribed biperiden were found to have a higher number of drug interactions. Similar results have also been reported in a study conducted in Mexico. In that study, 240 drug interactions were identified among 80 patients, and it was shown that biperiden use was responsible for 40% of these interactions [40]. In addition to the inherent risk posed by psychiatric disorders, our findings revealed that pharmacodynamic interactions leading to enhanced anticholinergic effects were highly prevalent among psychiatric inpatients with mean ACB and ADS scores reaching notably high levels (5.56 ± 3.32 and 4.15 ± 2.79, respectively). Recently, a study demonstrated that a vast majority of psychiatric inpatients are exposed to significant, sustained, and clinically meaningful levels of anticholinergic burden primarily driven by antipsychotic polypharmacy and concomitant medication regimens [41]. Although long-term cognitive outcomes or dementia incidence were not measured in our study, substantial epidemiological literature suggests that high exposure to drugs with elevated ACB scores, alongside severe underlying psychiatric conditions, may be associated with long-term neurocognitive decline [42,43]. Clinicians should maintain a careful risk–benefit balance. In clinical practice, the utilization of biperiden represents a critical therapeutic balance; while inadequate control of extrapyramidal symptoms can severely worsen a patient’s functional status, reduce treatment adherence, and lead to poorer psychiatric outcomes, its use concurrently exacerbates the overall anticholinergic burden. Close attention may need to be paid to the concomitant use of other potent anticholinergic medications that could further accumulate burden.

The findings of this study identify important directions for future research. Further prospective studies evaluating medication regimen from pre-admission through discharge, including medications added, discontinued, and dose-adjusted during hospitalization, would provide more understanding of the importance of drug use evaluations in the early period of admission. It may support the development of new scales for prescribing patterns and hospitalization outcomes.

### Limitations

Several limitations of our study should be considered when interpreting the results. Primarily, the retrospective and single-centre nature of the research may limit the generalizability of our findings to broader clinical settings or different patient populations. Additionally, our assessment of medication use was restricted to prescriptions issued within the first 72 h of hospitalization, thereby excluding pre-admission chronic treatments and post-discharge medication regimens. This focus might have constrained our ability to evaluate the full continuity of therapy and its long-term outcomes; therefore, further multicentre, prospective studies are needed to validate these observations across the entire care continuum. The psychiatric categories utilized in this study reflect primary clinical presentations and reasons for acute admission rather than mutually exclusive, long-term diagnostic classifications. Because overlapping clinical features are common in acute settings, as evidenced by the co-occurrence of both psychotic and mood symptoms in a subset of our patients, separate subgroup analyses comparing monotherapy versus polytherapy within rigid diagnostic boundaries were not feasible and could limit the specificity of findings for single isolated disorders.

## 5. Conclusions

Our study shows that antipsychotic polypharmacy within the first 72 h of admission independently predicts longer hospital stays. We also found that psychiatric inpatients had a high anticholinergic burden, and patients receiving antipsychotic polypharmacy experienced notably more potential drug–drug interactions. Detecting antipsychotic polypharmacy early may help clinicians identify patients at higher risk for prolonged hospitalization and those needing closer medication management. Psychiatric polypharmacy may be a clinical necessity in complex and difficult-to-treat patient groups. However, it should be remembered that it requires careful individualization and close monitoring. Systematically reviewing drug use in the first 72 h can help assess medication burden and improve overall medication management. Early clinical pharmacy interventions, such as medication reviews, anticholinergic burden monitoring, and drug–drug interaction screening, may be crucial for optimizing pharmacotherapy and improving patient outcomes in psychiatric inpatient settings.

## Figures and Tables

**Table 1:** Demographic and clinical characteristics of the patients (*n* = 280).

Characteristic		*p*-Value
**Sex, *n* (%)**		
Female	106 (37.9%)	
Male	174 (62.1%)	
**Age (years), mean ± SD**	38.65 ± 13.86	
Female	37.83 ± 13.66	0.209
Male	39.98 ± 14.16	
**Age Categories, *n* (%)**		
<65 years	267 (95.4%)	
≥65 years	13 (4.6%)	
**Reasons for hospitalization, *n* (%) ^a^**		
Psychotic disorders	137 (48.9%)	
Mood disorders	124 (44.3%)	
Anxiety disorders	31 (11.1%)	
Substance use disorders	21 (7.5%)	
Personality disorders	18 (6.4%)	
Intellectual disability	9 (3.2%)	
Other	21 (7.5%)	
**Co-morbidities, *n* (%)**		
None	220 (78.6%)	
Hypertension	24 (8.6%)	
Diabetes mellitus	22 (7.9%)	
Thyroid disease	16 (5.7%)	
Hyperlipidemia	8 (2.9%)	
Asthma	4 (1.4%)	
Cerebrovascular disease/TIA	3 (1.1%)	
Other †	6 (2.1%)	
**Length of stay (days), mean ± SD**	21.66 ± 12.14	

† Other comorbidities include dementia (*n* = 2), peripheral vascular disease (*n* = 1), heart failure (*n* = 2), COPD (*n* = 1). ^a^ Patients could be assigned multiple reasons upon admission.

**Table 2:** Drug utilization characteristics.

	*n* (%) or Median (IQR)
Total number drugs per patient	4 (3–5)
Polypharmacy	106 (37.9%)
Number of patients on antipsychotics	263 (93.9%)
Number of patients with antipsychotic polypharmacy	179 (63.9%)
Number of patients on antidepressant	88 (31.4%)

**Table 3:** Most frequently prescribed drug classes other than antipsychotics and antidepressants.

ATC Code	Drug Class	Prescriptions	Patients *n* (%)
N03AG	Antiepileptics (valproic acid)	66	66 (23.6%)
N04AA	Anticholinergics (biperiden)	65	63 (22.5%)
N05BA	Anxiolytics (lorazepam)	58	57 (20.4%)
A11DB	Vitamin B combinations	45	45 (16.1%)
A02BC	Proton pump inhibitors	33	33 (11.8%)
H03AA	Thyroid hormones	19	17 (6.1%)
N03AF	Antiepileptics (carbamazepine)	16	16 (5.7%)
B01AC	Platelet aggregation inhibitors	16	16 (5.7%)
C07A	Beta-blockers	19	19 (6.8%)
C09A/C	ACE inhibitors/ARBs	14	13 (4.6%)

**Table 4:** Most frequently identified drug–drug interactions by risk category.

Drugs	Risk Category	*n*	Potential Clinical Effect
Olanzapine-Quetiapine	C	78	Seizure threshold lowering/QTc prolongation risk
Quetiapine-Risperidone	C	67	Seizure threshold lowering/QTc prolongation risk
Quetiapine-Valproic acid	C	37	Enhanced CNS depression
Olanzapine-Valproic acid	C	31	Decreased olanzapine concentration
Biperiden-Quetiapine	C	30	Enhanced anticholinergic effect
Haloperidol-Quetiapine	C	30	Seizure threshold lowering/QTc prolongation risk
Clozapine-Amisulpride	C	20	Enhanced hypotensive effect
Olanzapine-Lorazepam	C	18	Enhanced CNS depression/olanzapine toxicity
Biperiden-Olanzapine	C	17	Enhanced anticholinergic effect
Biperiden-Risperidone	C	16	Enhanced anticholinergic effect
Clozapine-Biperiden	D	9	Enhanced constipating effect of clozapine
Risperidone-Carbamazepine	D	8	Decreased risperidone concentration (via CYP3A4 induction)
Carbamazepine-Quetiapine	D	7	Altered carbamazepine/quetiapine concentrations
Lorazepam-Valproic acid	D	7	Increased lorazepam concentration
Clozapine-Quetiapine	D	5	Enhanced constipating effect of clozapine
Sulpiride-Olanzapine	X	2	Enhanced sulpiride toxicity
Quetiapine-Chlorpromazine	X	1	QTc prolongation risk
Antipsychotics-Ipratropium bromide	X	8	Enhanced anticholinergic effect

**Table 5:** Factors associated with the number of DDIs.

Factor	Median (IQR)	*p*-Value
Polypharmacy (−), *n* = 174	2 (1–4)	
Polypharmacy (+), *n* = 106	7 (5–10)	**<0.001**
Antipsychotic polypharmacy (−), *n* = 101	1 (0–2)	
Antipsychotic polypharmacy (+), *n* = 179	5 (2–8)	**<0.001**
Biperiden (−), *n* = 217	3 (1–6)	
Biperiden (+), *n* = 63	6 (2–10)	**<0.001**

**Table 6:** Anticholinergic burden scores by antipsychotic polypharmacy.

	All Patients (*n* = 280)	No Antipsychotic Polypharmacy (*n* = 101)	Antipsychotic Polypharmacy (*n* = 179)	*p*-Value *
ACB score, mean ± SD	5.56 ± 3.32	2.95 ± 1.53	7.03 ± 3.15	**<0.001**
ADS score, mean ± SD	4.15 ± 2.79	2.08 ± 1.46	5.32 ± 2.68	**<0.001**
ARS score, median (IQR)	2 (1–3)	1 (0–2)	3 (2–3)	**<0.001**

* Student’s *t*-test for ACB and ADS; Mann–Whitney U for ARS.

**Table 7:** Bivariate comparisons of length of hospital stay by clinical factors.

Factor	*n*	Mean ± SD	*p*-Value
Comorbidity (−)	220	21.41 ± 12.50	
Comorbidity (+)	60	22.57 ± 10.79	0.515
Polypharmacy (−)	174	20.61 ± 11.72	
Polypharmacy (+)	106	23.39 ± 12.68	0.063
AP polypharmacy (−)	101	19.23 ± 11.64	
AP polypharmacy (+)	179	23.03 ± 12.24	**0.012**

**Table 8:** Negative binomial regression model for length of hospital stay.

Variable	Coef.	IRR	95% CI	*p*-Value
Intercept	2.682	14.615	11.176–19.112	**<0.001**
Age (years)	0.006	1.006	1.001–1.012	**0.019**
Sex (male)	−0.030	0.970	0.842–1.118	0.678
Medical comorbidity	0.023	1.023	0.856–1.223	0.800
Antipsychotic polypharmacy	0.190	1.209	1.044–1.400	0.011
Psychotic disorder	0.157	1.170	0.992–1.380	0.062
Mood disorder	−0.124	0.883	0.749–1.042	0.140

IRR: incidence rate ratio; CI: confidence interval; Model AIC = 2152.4, BIC = 2181.5. Dispersion parameter α = 0.275.

**Table 9:** Summary table of key findings.

Finding	Clinical Implication
AP polypharmacy → 20.9% longer LOS (IRR = 1.209, *p* = 0.011)	Early antipsychotic review may predict length of stay
88.9% patients had ≥1 DDI	Systematic DDI screening is warranted at admission
High anticholinergic burden in AP polypharmacy	ACB assessment should be integrated into medication review

## Data Availability

The datasets used and/or analyzed during the current study are available from the corresponding author on reasonable request.

## Supplementary Materials

The following supporting information can be downloaded at: https://www.mdpi.com/article/10.3390/medicina62061063/s1, Table S1: Univariate Negative Binomial Regression Results for Candidate Variables.
