# Supplementary material for: Drug Utilization, Anticholinergic Burden and Predictors of Length of Stay in a Psychiatric Hospital: A Retrospective Observational Study
Source: Medicina (Kaunas). 2026 May 31;62(6):1063. doi: 10.3390/medicina62061063 (PMC13304261; doi:10.3390/medicina62061063)
Supplement: Supplementary file 1 [file medicina-62-01063-s001.zip › medicina-4293150-supplementary.pdf]

Supplementary Table S1: Univariate Negative Binomial Regression Results for Candidate Variables

Variable	IRR	95% CI	p-value
Age (years)	1.006	1.001–1.011	0.019
Sex (male)	1.006	0.877–1.153	0.936
Medical comorbidity	1.054	0.897–1.238	0.524
Total drug count	1.026	0.997–1.055	0.077
Polypharmacy	1.135	0.990–1.301	0.070
AP polypharmacy	1.198	1.043–1.376	0.011
Biperiden use	1.153	0.984–1.350	0.078
Psychotic disorder	1.291	1.131–1.475	<0.001
Mood disorder	0.830	0.722–0.954	0.009
